# Zoom Out Camera! The Reflexive Character of an Enactive Account

**DOI:** 10.3389/fpsyg.2020.00919

**Published:** 2020-05-06

**Authors:** Fred Cummins

**Affiliations:** UCD School of Computer Science, University College Dublin, Dublin, Ireland

**Keywords:** enaction, laws of form, ecological psychology, reflexivity, adaptivity

## Abstract

The reflexive character of enactive theory is spelled out, in an effort to make explicit that which is usually implicit in debate: that we are responsible for the distinctions we draw, and that ultimately, the world that we collectively characterize is a joint production. Enaction, as treated here, is not a positivist scientific field, but an epistemologically self-conscious way to ground our understanding of the value-saturated lives of embodied beings. This stance is seen as entirely congruent with the scientific field of ecological psychology, which is itself then cast as a specific example of the kind of science that can be done in an enactive mode.

## 1. Introduction

At the end of Alejandro Jodorowsky's cult film The Holy Mountain, the pilgrims have shed themselves of their worldly attachments, have ascended the mountain, and there is every expectation that enlightenment, or some form of spiritual elevation will be found at the summit. Instead, one of the figures at the table on top of the mountain turns out to be the director, and he turns to face the camera. “Zoom out camera!” he instructs unambiguously, and as the camera pulls back, we reinterpret the whole situation as a group of actors on a set. We see the boom microphones, the props and the make-up assistants, all the trappings of the making of a film. Conventions are made to be broken, and the provocation or perturbation that happens to the audience when the fourth wall is broken like this has been played with since the Proscenium Arch was first erected.

One effect that can arise is that the viewer is solicited to enter more fully into the fictional world, as when the player of a video game is encouraged by an on-screen character to have the player's avatar engage in the action. Far from placing the player outside of the fiction, this serves instead to enlarge the magic circle, so that the player is now, to some extent, in the world of the fictional beings (Conway, 2010). When Kevin Spacey winks at the camera in House of Cards, it is likewise to include the viewer as a co-conspirator, not to negate the drama.

Another effect that might happen is disenchantment or alienation. When the audience is directly addressed, the fiction is unmasked as nothing more than fiction. They must abandon the pleasant conceit and face a stark reality. This kind of cold shower for the imagination was used by Bertolt Brecht as a way of directly infusing urgent political content into the distraction of the theatrical play. After disenchantment, there is no pretending any more. This is what Jodorowsky does too, though for rather different purposes. “What?” he seems to say. “You expected enlightenment from a film? Pick up your own damn cross and carry it!”

Jodorowsky's challenge to the viewer resembles the challenge that an enactive account presents to those who engage with it. The injunction this time might better read “You expected an account of reality from a model or a story? Own your own distinctions!” Let's explore some of those distinctions.

## 2. Two Accounts of the Enactive Cell

The most frequently presented exemplary embodiment of the central concerns of the enactive literature is the wistful picture of a lone cell in a petri dish, equipped with a single glucose sensor hooked up to its means of propulsion, and mechanically locomoting toward a distant nutrient source (Cummins and De Jesus, 2016). In picking out one direction rather than another, the cell is drawing a distinction. We need to develop an awareness of the difference between the distinction the cell is drawing (self/non-self) and the distinctions we draw (picking out a cell, a source, a medium, etc.). The cell knows nothing of chemistry or geography, but is, as far as we can tell, pursuing the project of its own continued existence. To us, the cell appears to be acting in its own interests by swimming toward a nutrient. In the example, glucose is taken to play a role in the metabolic economy of the cell, and the business of detecting the ambient glucose gradient, and hence navigating toward the source, pretty much exhausts the sense-making activity of the cell. Sense-making, here, is the regulated exchange with the surrounding petri dish that the cell conducts in order to persevere as a distinguishable unity, as, indeed, a cell.

Why is this picture redrawn, again and again? In Cummins and De Jesus (2016) we explored this central narrative myth in detail. We listed Maturana and Varela (1987, p. 148/149), Thompson (2007, p. 74), Barandiaran et al. (2009), Froese and Di Paolo (2011), and Egbert et al. (2010). Many more examples could have been adduced, and the chemotactically swimming cell continues to thrive in the literature, if not in the petri dish. Formally, the model cell, as we describe it, illustrates no more and no less than a first order cybernetic system, not terribly different from a thermostat or a heat-seeking missile. Such a system can be understood as a concrete embodiment of an abstract notion of control, provided by the set point to which the system converges through negative feedback with its environment: for the thermostat, that is a temperature that lies within specific bounds which are determined by the user; for the missile, it is a trajectory that converges on a pre-specified target selected by the programmer. What is the equivalent of the temperature or the target for the cell? If we are to accept the claims of the enactive literature it is the cell's own persistence as a dynamically individuated entity that we are observing. Unlike the thermostat or the missile, this purposiveness is understood by us to originate with the cell itself, to be emergent, expressing the perplexing “natural purposes” of the living organism (Weber and Varela, 2002). Here, we should feel the 4th wall straining, as we become aware that our own recognition of the cell as a dynamically individuated entity cannot be separated from any account of the cell as a unity. Our attention is captured by the cell as a minimal form of life, persisting in the only way it can, asserting itself in its sense-making. By contrast, in the domain of observation that we bring forth, the cell is a mechanism that swims toward the glucose source, with apparent purpose. This is a disenchanted account, of the kind science likes to construct, but it is not a simply objective account. It is teleonomic in form^1^, in that it attributes an “as-if” purposiveness to the cell, allowing the cybernetic characterization. It is an account that we have framed by drawing specific distinctions. It is objective, but an objectivity in parentheses, as Humberto Maturana frequently puts it (Maturana and Poerksen, 2004). We can see our own role in bringing forth the domain of observation by pulling back to become aware of the ground from which we draw distinctions.

One obvious reason for the centality of the account of the lone cell is that contemporary enactive theory draws inspiration from the theory of autopoiesis associated primarily with Maturana and Francisco Varela (1980). Autopoiesis was, and is, a hugely influential account of what it means to be a living being, and it was articulated in the chemical domain of the cell, with due regard for the complexities of biochemistry. Although some have tried to retrospectively identify an “autopoietic” school of enactive thinking (Hutto and Myin, 2013), the term “autopoiesis” did not appear in the volume “The Embodied Mind” (Varela et al., 1991), usually understood to be an original source text from which enactive theory draws, and autopoiesis is and remains a theory of philosophical biology, not a general theory of enaction. This distinction has often been blurred, to the point of eradication, as theoreticians concerned with the characterization of autonomy in terms of operational closure have chosen to describe many kinds of biological and social systems as autopoietic (Luhmann, 1995; Hutto and Myin, 2013). This genie can probably not be put back into the bottle, and we will have to contend with the confusion of autopoiesis and autonomy in the future. But there are some very important characteristics of the account of the cell, developed in autopoietic theory, that go on to inform and shape all enactive theory. I will draw some of them out as best I can, seeking to situate the concerns of enactive theory in a broader landscape of motivated theorizing. The reader is warned that the niceties of distinction drawn here are not always presented in the same way or with an identical concern in the enactive literature.

### 2.1. The Domain of Operation

The cell exhibits *operational closure*. This means that we understand the cell to consist of a set of circularly linked processes of production, which we might caricature as A producing B producing C …producing A^2^. The processes produce something, of course, and what they produce are the components required to keep the processes of production going, and of course the boundary that contains all those processes. This is the recursive character of the self-producing processes within the domain of operation of the cell: they produce themselves, and in so doing, the unity perseveres from one moment to the next. These processes are not a perpetual motion machine, of course, and so some form of energy supply is needed to keep the whole affair ticking over, along with some means of getting rid of waste. The cell thus engages in regulated exchange with its surround, through careful upkeep of a border, the membrane, across which substances may be taken in or released. However, in the domain of operation of the cell, there is no representation of an exterior, and there is no appeal to purpose, goal, or function, even in the teleonomic sense. The register within which the processes are described is deanimated, free of any tinge of agency. There is simply the ceaseless churn of self-production, and when it stops, so too does the cell as a living entity. In this way, Maturana and Varela describe the autopoietic unity as a “machine.”

### 2.2. The Domain of Observation

We now need to pull back and consider the cell in its immediate physical and chemical context. Having recognized the cell as a bounded self-producing unity, we can now distinguish it from other elements that we see around it. We, as observers, can see the mindless churn of the cell as achieving self-production and self-distinction simultaneously (Di Paolo et al., 2018). In the case of the cell, the membrane itself makes clear (to us) the separation between cell and non-cell. The cell/non-cell distinction is spatial, bounded, and easy to point out. If we move on to consider other autonomous unities, this is work that would need revisiting. As we consider the cell in its environment, we will need the teleonomic frame we met above to characterize the cell as a cybernetic system. This is because the activity of the cell becomes more intelligible to us, as observers for whom it is a distinguished self-producing unity, if we speak of the “function” of glucose in its metabolic economy, or the “function” of its movement as being the acquisition of nutrients. Function reflects purpose. Functional ascription is thus available to us in the domain of observation, but not in the domain of operation. Did we just open a chink to unbounded teleological explanation of the kind associated with Aristotle, and shunned by latter-day science? No, but the charge has to be taken seriously.

I will here simply adopt the position of Varela (1979, p. 73) who points out that the two forms of explanation, operational and observational, are both to be understood relative to the perspective of some observer(s)^3^. Our hypothetical observers might hope to exhaustively characterize the steps within the domain of operation, itemizing reactants, catalysts, and products, measuring reaction rates and quantifying ingredients and products at each step in the circular chain of production, and thus arrive at a naturalized, causal, account of goings on. This seems to be a reasonably finite task (cell biologists may demur, but we are concerned here with matters of principle alone). In contrast, understanding the causal chain that gave rise to this particular form of organized matter, and its structural coupling to its surround, seems to be an impossible task, even in principle. The ascription of function or purpose then is to be understood as a form of shorthand, “conceptually abbreviating the intermediate steps of a chain of causal events, and concentrating on those patterns that are particularly interesting to the inquiring community” (Varela, 1979, p. 73). Teleonomy is not teleology, and the appeal to function serves to make things intelligible to the observers, who have chosen to make specific distinctions.

In laying out things in this way, Varela has introduced a crucial semiotic distinction between the domains of causal and of symbolic explanation, motivated by the explanatory requirements of a specific observer, or, more importantly, a community of observers—for explanation is a public act. A fully causal, or nomic, account of the cell would require the inclusion of an uncountable number of variables, interacting over millennia, nay billions, of years, and would thus not satisfy the demand for explanation of any community. But not everything in that history is relevant to the community's intentions in regarding the cell as indicated by them, and those aspects of the cell that are of interest are well-accounted for by ignoring the causal chain and describing the resulting relationships with recourse to teleonomic terms.

Varela expands on this thus:

The possibility of choosing to ignore intervening nomic links is at the base of all *symbolic* descriptions. What is characteristic of a symbol is that there is a distance, a somewhat arbitrary relationship, between signifier and signified. This is, of course, very immediate in human discourse: Words and their contextual meanings have such a remote and involved historical and structural mode of coupling that any effort to follow such nomic connection is hopeless (Varela, 1979, p. 73).

## 3. Of Indices, Icons, and Symbols

The introduction of the notion of distinct causal and symbolic forms of explanation opens the door immediately to semiotic issues, and we might feel it necessary to ask about the processes of elision, compression, selection, and exclusion that are assumed to underlie the teleonomic, or symbolic, account we provide in the domain of observation. The confident assertion that we might arrive at an acceptable explanation (for a specific community) with free use of teleonomy, without thereby introducing the bugaboo of teleology, requires some careful consideration. It is virtually axiomatic in the hard sciences (physics, inorganic chemistry) that an undisciplined appeal to function or purpose turns any account into mere wishful thinking, while functional ascription is riotous and exuberant in the psychological and social sciences. Ecological and biological fields have their own specific reliance upon certain kinds of functional description. A biology of the organism is unthinkable without appeal to function, but an ecological picture becomes more complex as the environment turns out to be constituted by and constructed by other living agents. Neo-Darwinian accounts of evolution typically eschew any appeal to function. Care is needed here, and the territorial issues that arise run deep. In particular, it would behove us to be as aware as we can be of the frame of any specific discussion, with its attendant unwritten commitments, and its selective delineation of entities and processes, which, in being discriminated, thereby bring a discursive landscape into being within which some things can be expressed, and others not.

A possible starting point to ground such discussion lies in the twilight domain of human somatic physiology, with one foot securely in biology and the other steeped in human values and normativity. Here we might hope to refine our use of terms, and to prepare the ground for subsequent discussion in more contentious domains. We can take as our anchor, as the hinge on which everything turns, our shared understanding we (as a collection of observers, including you, the reader) have for the importance of the integrity of the individual human body. It requires little debate to agree that we need stories in which the continued persistence of the human living body features as a central element. Such accounts may, perhaps, be teleonomic, but they are no less secure for all that, precisely because we share this common ground, this unstated and inalienable interest in the integrity of the human body. We usually do not have to argue this, or better, if we adopt a stance toward the body that does not presuppose this singular value, we will immediately alienate other people with whom we jointly observe, describe, and discuss things (different conversations may develop from different starting points, with different framing considerations, and different attachments to obligatory value commitments).

Given this common ground, it is now unproblematic to provide two distinct, but non-contradictory, accounts of what the heart is doing. In the domain of operation, we will observe a complex unity composed of several cell types that displays gross collective movement organized as a regular pulsation by means of an appropriate distribution of nerve cells. In this operational description, there is no place for any appeal to function. We ask what are the parts of the heart, and how do they interoperate. The heart, in this account, is a machine.

When we pull back and view the heart in its larger context, we are drawn inexorably to consider its position as one organ in a larger composite whole, the human body, in which we have a concerned interest. Note that we could have chosen other frames of reference, other contexts, within which the pulsation of the heart would be a contingent detail, playing no organizational role. However, in adopting the frame of physiological integrity, we now see the heart as a pump. Its role in this context is to pump blood, and the circulation of blood is an integral part of the sense-making activity of the body considered as a whole as it perseveres from one moment to the next.

The importance of these framing considerations that allow us both a mechanical and a teleonomic account of the heart is made clear by Stafford Beer in his preface to Maturana and Varela (1980): “Note that Aristotle thought that the brain was a ‘human radiator,' namely an apparatus for cooling the blood. Note also that he was right” (Maturana and Varela, 1980, p. 68). Framing is, in a sense, everything.

Varela describes the teleonomic account as “symbolic” because the continuous causal chain of a fully nomic account has, of necessity, been interrupted in order to discuss the unity as it appears to our curious gaze. This introduces a possible challenge to any scientific account that leans on such a description, should the abbreviations and simplifications that lie between the teleonomic account and a strictly causal account be refused by the collection of observers. This tension is an old one, long predating the theoretical biology of autopoietic or enactive theories. To examine this a little further, I will make use of the tri-partite distinction between index, icon and symbol most commonly associated with Charles Pierce, though my interpretation of these three key terms will be my own. All three terms speak of an intentional relation between a sign of some sort, and the grounded incarnate reality of which the sign speaks. The sign of which we here speak is an element within the domain of description, whether it be operational or observational, for all descriptions must employ words, symbols and the like. We might take the situated collective discourse as an interpretant.

An *index* arises when there is a causal link that we can follow exhaustively^4^. This is typically conceived of as a single step in mediation. The body contracts a disease, and spots appear on the skin. The spots stand in a causal relation to the pathology and allow confident inference about the underlying condition. The spots are an index. A polaroid photograph of a scene might likewise be considered an index, as we can follow all causal steps from the photo in our hands back to the distribution of light in the original scene (more elaborate forms of photography complicate things precisely because of representational hiatuses that are introduced and the complex embedding of the original registration in distributed patterns of activity, e.g., digital storage on computers).

A *symbol* stands in a purely conventional relation to the world. Causal links are not traceable in any complete sense. A symbol might be replaced by another and, if the convention be adopted, the symbol-referent relation will be preserved.

Between these two extremes lies the *icon*, and here I depart more radically from Pierce's account somewhat to adopt a more expansive view of icons as they have featured throughout millennia in religious wars and periods of iconoclasm, in which the topic of heated disagreement (real wars!) lies in the interpretation of the links between the descriptive element or sign (an icon) and its presumed referent. Pierce relies on a notion of “similarity” between icon and its referent, but the notion of similarity turns out to be untrustworthy, as well-argued by Goodman (1976). I will instead ask about the chain we must follow to trace a path from sign to referent.

To a member of one of the Orthodox churches, which place a high value on icons as objects of veneration, an icon is not an arbitrary picture. It arises within a tradition, and the process of icon production carefully guards against free invention and whimsy at every point within that tradition. Innovation does occur, of course, but it is strictly controlled, and acceptable only when introduced by those steeped in the tradition. An icon is typically a copy of another icon, and the lore of the tradition traces specific icons back through a chain of careful copies to some original. Of course the view of what an “original” is must be left to those who lie within the tradition, but in an idealized form (equivalent, perhaps, to the physicist's spherical cow in a vacuum) we might consider an origin in an acheiropoieton, an image not made by human hands, such as the image of Christ's face imprinted on the towel of Veronica, or the shroud of Turin, which has been claimed to be a direct imprint of Christ's body in the tomb. The first image, the acheiropoieton, is index-like, in that it stands in a causal relation to the original. Within the icon tradition, copies made from such an index likewise stand in a chain, and the chain extends all the way to the situated present. Of course this is not a causal chain in the sense implied by a naturalized account, but the chain is not arbitrary either, and the theoretical possibility of tracing the intervening steps is central to the structure of the icon making tradition. This may be a matter of faith, but it is a faith of the kind we ourselves display when we regard the steps of mediation as unproblematic that would serve to bring the operational and observational accounts into alignment.

Iconoclasts object. The reasons for objection are inevitably mired in the political discourse of the day, and the terms of the debate, and its resolution, will differ from one instance to the next. It is not necessary here to tease out fine distinctions between worshipping an image or statue and merely venerating it, though such debates have been important in allowing iconic traditions to persist into modernity. The core of the iconoclasts' objection is, however, very similar to the disenchantment that arises in scientific accounts when we adopt the strongly objectivist stance of the hard sciences and bar any appeal to function. The trust in an unbroken chain, it is asserted, is misplaced. The hiatuses are unbridgeable.

Varela asserted above that any effort to follow the nomic relations that gave rise to a specific embodied organization we observe and describe is hopeless. This is the practical impossibility outlined also in Cummins and De Jesus (2016) who considered the limitations of a first order cybernetic sketch of the enactive cell as so often presented in the literature. The cell appears as an already-formed unity whose originary story is not miraculous, but is also not something we can make perfectly explicit, as doing so would require simultaneous description of the entire history of the organism, and its sociality, that is, its history and the history of everything it had interacted with and everything those things had interacted with, and so on until we are forced to draw the entire history of the cosmos into our account. There is no obvious limit to the degree to which the contingencies of history and prior interaction (and the prior historical trajectories of each of the interactants) are relevant to the present observations. Put baldly: some assumptions are going to be necessary if we are to assert anything at all of substance, or, to paraphrase Richard Feynman: In order an answer any why question, we much be within some frame of reference, in which some things are simply allowed to be.

Varela is asking us, as embodied beings, to join the faithful. The faithful here is merely the set of embodied beings who try to understand their world and its inhabitants, from singular perspectives rooted in the unity of the personal body. The trust that is required is that it might be possible, in principle, to move from the teleonomic observational description to the mechanical operational description, even if such a move is impossible on practical grounds. The reasons we might be willing to join him is the logical coherence of the framing which makes the activity of the living intelligible to us, and the desire to develop accounts in which the singular perspective associated with the individual body is a central anchor. The consensus we arrived at with respect to the heart provides grounds that we might cautiously grow such accounts to better understand what it is to be an embodied being. No guarantees are provided.

## 4. The Innovation of Adaptivity

Adaptivity was introduced in Di Paolo (2005) as an attempt to bridge the link between an operational (mechanistic) account of the cell and an observational account (teleonomic). It was noted that the strict separation of the two accounts left an explanatory gap, as there was no obvious way in which the operational description of a cell could take into account operating conditions in which the continued flourishing of the cell was threatened by external conditions. The proposed additional concept of adaptivity to the vocabulary of enaction sought to allow a view of the cell as *self-regulating* with respect to the boundaries of its own viability. Absent this additional concept, the activity of the cell could not be properly viewed as sense-making.

Di Paolo identifies two separate normative dimensions that call out for recognition in an account of a living unity. The first is the norm exhibited by an autopoietic entity, resulting in the continued process of self-maintenance and self-distinction. The second is the norm of sense-making, which must, it seems, appeal to homeostatic (i.e., cybernetic) concerns and is thus not available in the purely operational terms provided by autopoietic theory. I suspect that Di Paolo is correct in his view that some notion of self-regulation is necessary to understand the living being as a locus of self-concern, and that this does not follow from the strictly causal account provided by the domain of operation.

The norm generated by autopoiesis, which produces and distinguishes the unity simultaneously, allows us to recognize when it has been violated: the cell is dead. As a norm, this must be apportioned to the domain of the observer, for it was we who chose to pick out the cell as a unity. The norm that distinguishes existence from non-existence never played any role in the domain of the operation of the now deceased cell. In this respect, we might also recognize a norm arising from the suite of atmospheric and topographical processes that bring into being a tornado. Here too, if we have chosen to identify a tornado as a dynamically individuated entity, we can distinguish between situations in which it exists, and when it goes away again. There is no suggestion I am aware of that a tornado actively regulates the condition of its own existence, and if one were to characterize any such activity, it might be viewed as likely to succumb, upon further study, to a purely nomic account, devoid of any teleonomic terms. But tornadoes belong in a different conversation. We are here concerned with unity and autonomy as exhibited by the living.

Crucially, there is more than mere existence as a dynamically individuated entity at stake here. With respect to the discursive exemplar of the chemotactically ascending cell, Di Paolo notes “Bacteria will not seek higher concentrations just because they are autopoietic, since improving the conditions of self-production is not part of the definition of autopoiesis” (p. 437). Rather, self-production, in this case, requires that encounters with the world are “evaluated” by the system in as much as they contribute to the maintenance of autopoiesis. In the absence of such evaluation, it would appear that an autopoietic account might preclude any satisfactory account of stress, illness or fatigue, for the cell is not provided with any means to notice or respond to changes in its environment that do not actually kill it. This is an important lacuna for any biological theory of the organism.

However if we come back to the strictly separated domains of operation and observation, we find that the mechanical characterization of the cell that is repeated again and again provides almost all the required machinery to carry out this “evaluation” in a strictly non-teleological manner, for the cell as described is not just a collection of metabolic processes, but it is conventionally described as having means for registering the ambient glucose concentration over time, and means for allowing that determination to modify the probability of switching from a random to a directed mode of locomotion and back again. This is the first order cybernetic machine we noted above. Augmenting this mechanism with a graded response to ambient glucose concentration provides a similarly mechanical implementation of adaptivity. It is a carefully drawn sketch of what it is to be sensitive to something. As with any such machine, the goals that we recognize are external, as imposed by the designers of the machine, which is us as we make distinctions in the domain of observation.

What seems to be missing here is the assent of the community of observers that the teleonomic description could, in principle if not in practice, be unrolled into a much larger nomic account, without any fanciful inventions. This is the leap required of the observing community by Varela in order to “conceptually abbreviat[e] the intermediate steps of a chain of causal events, and [to] concentrat[e] on those patterns that are particularly interesting to the inquiring community” (Varela, 1979, 92). In Cummins and De Jesus (2016) an argument was made that two essential elements were missing from the portrait of the enactive cell that must be included in any account of any living organism: historicity and sociality. Each of these precludes any exhaustive reckoning for any real case, making the causal chain strictly untraceable, leaving us with the necessity of evaluating, for ourselves, the plausibility of the leaps and bounds required to produce the cell we see. The mechanical elements that do the heavy lifting, steering the cell toward its nutritive source, have been drawn by us, on the shared understanding that the cell is not merely alive, but self-regulating.

Reliance on the untestable assumption that the two domains of observation may be reconciled in principle if not in practice may look worryingly like a leap of faith, rather than a strongly objective scientific programme. I want to suggest that this is not quite accurate, it is not necessarily a problem (though it demands being taken seriously), that it opens the way toward a scientific epistemology grounded in the embodied concerns of the living, and that the need to introduce adaptivity to such an account demarcates an important development in enactive theory that takes it beyond autopoiesis, and has consequences for the further development of such theory.

## 5. Of the Question of Naturalization

I have deliberately employed scandalous terms, such as “faith” and “faithful” as a counterweight to the repeated use of the terms “science” and “nature,” “natural” and “naturalization” as they have been wielded in the enactive literature, taking particular note of their use in motivating the innovation of adaptivity in Di Paolo (2005). I do this to draw out the necessary tension that arises around what might be considered a “naturalistic” account. The term “naturalization” is wielded most often when the concepts in play seem to be of dubious ontological status, relying on assumptions that have been freely invented, rather than painstakingly induced through observation. Thus, for example. Barandiaran (2017) asserts that theories of autonomy provide a “naturalized account of normativity,” Di Paolo, drawing on Jonas, discusses a “naturalization” of teleology, and asserts that the suite of fundamental notions that enactive theory has received from autopoietic theory needs to be augmented with the notion of adaptivity in order to “naturalize sense-making” (Di Paolo, 2005), which in turns leads to considerations of “natural agency.” Weber and Varela (2002) provided a somewhat convoluted argument that sought to “naturalize teleology” without falling into the trap of reductionism. Behind the desire to naturalize our accounts stands the hope that the hiatuses that interrupt the symbolic account can, in principle, be unified with the continuities of a causal account, so that apparent teleology will be shown to be no more than mere teleonomy.

When we maintain care in our distinctions between the operational and observational domains, it is of paramount importance that we examine the extent to which we are relying on appropriate epistemological foundations. The reflexive self-awareness of the observer (community of observers) drawing a distinction is a central part of any enactive account, and this second-order cybernetic injunction precludes any unthoughtful appeal to a simply existing world. Rather, it requires that the distinctions drawn be uncontroversial for the observing community. This rules out unreflective reliance on notions of the physical (pre-existing? products of physical theory? or merely uncontroversial?), the objective, or even the natural. We can illustrate this by appeal, once more, to how the heart features in an operational and an observational account. It would not be unreasonable to describe William Harvey's influential account of the circulation of the blood (1628) as a naturalization of the role of the heart in the economy of the body. The account was not immediately accepted, but needed further argumentation and the test of public debate to come to its present role as a generally shared understanding. This consensus was possible as it was effective at making intelligible to a broad community how the heart *functions* given the framing context of the body's physiological organization. The 2-fold view of the heart is, I hope, not perceived to be at variance with the tenor of scientific accounts.

We might be reminded at this juncture of the traditional contrast between emic and etic accounts of the form of structured human behavior. This distinction was introduced by Ken Pike (1967) to bring coherence to our descriptions of many domains of human activity. The origin of the contrast lies in the relation of phonetics (etic) to phonology (emic). Phonetics deals with uncontroversial observables: muscles, spit, waveforms of vibrating air, ear morphology, and so on. Phonology is an abstract structural domain of discrete elements (phonemes) which, it is asserted by linguists of a certain stripe, constitute basic element of a language. One might adopt an iconoclastic position with respect to the abstract domain of phonology (Port and Leary, 2005), and yet merrily work alongside a phonetician, whose observations are grounded, at least potentially, in the secure space of physical (here, meaning only uncontroversial) measurement. Emic accounts require us to agree on the structure of a domain in terms agreed by specialist practitioners. Such an account is an insider account, drawn in terms meaningful to those who subscribe to the discursive frame. It co-exists with etic accounts which might be understood as those made by outsiders, or that are couched in terms accepted and presumed by all discussants, without prior commitments to the dimensions and distinctions of the emic story.

The emic/etic distinction has traveled far afield. The need to accommodate insider and outsider views of cultural forms of organization arises, for example in cultural anthropology (Harris, 1976), cross-cultural psychiatry (Marano, 1982), comparative legal studies (Morris et al., 1999), ethnomusicology (Alvarez-Pereyre and Arom, 1993), cross-cultural psychology (Triandis et al., 1993), and many other fields of comparative social science. This allows parallel descriptions of one and the same event to be couched, one drawing on distinctions accepted and required by insiders, and another that requires no such commitment. So, for example, an etic account of the Roman Catholic sacrament of the eucharist would document liturgical form, event sequences, historical development, aesthetic qualities, etc., while an emic account would note that the event of transubstantiation takes place at a particular moment in the ritual, after which the host is changed in substance. Emic and etic accounts can exist in felicitous parallel as long as such borders are clear, as Pike's own use of a church service and a football match remind us.

To many, autopoiesis seemed to open the way to finally arriving at scientific, objective, accounts of what it is to be a living being. Enactive theory has grown beyond the autopoietic characterization of the single cell, and has set its targets on multi-cellular entities, and their social and ecological domains. At stake here is the foundation of scientific epistemology for science as conducted by embodied beings in the domain of the living. The rush to subsume enactive accounts under a science that has itself not yet developed such an epistemological foundation seems to this author to run counter to the promise that enaction holds out. The cautious distinctions between causal and symbolic accounts, together with the insistence that the community of observers bear responsibility for the distinctions they draw, suggests that the sciences of the living might be better understood as rooted in the kind of reflexive care that enaction has to offer, rather that viewing enaction as a specialization within scientific discourse. For it is in the care of drawing distinctions that are adequate to the task of explanation to a specific community that enaction can provide a foundation for epistemology in which the tools, methods, and insights of science can flourish. This is a bold claim, but one that might merit consideration as we take stock of what role scientific argumentation will play in a future in which our own position within the biosphere is threatened, and an urgent need arises to reconsider what a truly ecological science, capable of understanding the interdependencies of the living, might look like.

In a recent volume, Latour (2017) lays out a cogent argument that the concept of “nature” has become an impediment to our understanding of ourselves, our world, and our practices. “Nature” has played several different and incompatible roles in all such accounts. On the one hand, it has been thought of in operational terms (to adopt our present conceptual vocabulary). It is the non-negotiable domain whose characterization must be free of normative claims, for this is simply how things are. On the other hand, nature has been conceptualized as pointing the way toward felicitous being in the world, set apart from artifice, corruption, pollution, human hubris, as we speak of the nature of a species, of human nature, or of 100% natural yoghurt. Nature, here is strongly normative. When prescription (normativity) and description (operational accounts) become confused and inseparably entangled, there is no way in which our common articulation of a shared understanding can be rendered apolitical. Embodied beings are beings with specific vested interests. A science done by and for embodied beings is never free from the negotiation of the collective whose joint observations, and consensual distinctions, provide its raw material.

Latour does not offer a simple substitute. By leaning on the provocative figure of Gaia (Latour, 2017), he introduces a view of the territory we live on as multiple, contested, and saturated with agencies we are only beginning to recognize, but with which we have to contend. Gaia is a muddle and a mess, not a unified causal domain, and the frequent misunderstanding of Gaia as some kind of self-regulating super-organism is absolutely not what is being proposed. There is no helmsman in charge of the whole. The shift that is required is from a thoroughly disenchanted deanimated world driven by inexorable Laws of Nature to recognizing that the biosphere is animated through and through, with different kinds of organizational unities interacting, each affecting and being affected by others, each constituting part of the environment for the other, so that the notion of “an environment” goes away entirely, consigned to the same scrapheap of legacy concepts, such as Nature, or even Human.

The familiar distinction of humankind separate from the natural world is as incoherent, in his view, as the idea that we can cleanly separate the cultural, symbolic domain from the deterministic and causal. Once more we are faced with the task of reconciling symbolic and causal accounts, but, under the new climatic regime, or as he puts it, after the ecological mutation, our task is not merely clarification and the sharpening of the distinctions we draw. Rather, it is a diplomatic task in which we construct our best objective accounts using the tools and methods of science, to learn how to co-exist with others on the only territory that is available to us. An objective account, in this framework, is not the pristine transparent representation of an immutable truth, but an account that can withstand objections. This was the lesson William Harvey had to learn too.

Ecology clearly is not the irruption of nature into the public space but the end of “nature” as a concept that would allow us to sum up our relations to the world and pacify them (Latour, 2017, p. 36).

The emic/etic distinction is importantly different from a contrast between causal and symbolic accounts. Emic accounts make sense to insiders, whether they be phonologists, economists, or shaman. Etic accounts make use of uncontroversial observables (for a specific community), but they are necessarily prior to the commitment to any specific emic interpretation. Yet in considering our position among the living, we are all, to some extent, insiders. There is no outside vantage point we can adopt from which to illuminate an external reality. There is no outside. We are here. The process of pulling back, enlarging the frame to include our own commitment to distinctions that make our observations intelligible, goes on indefinitely (Nagel, 1986). Autopoiesis kicks things off, by providing a specific account of what the unity is that we see as a cell. Enactive theory goes further, not by changing its origins, but by drawing further distinctions as different kinds of unity are considered. As the questions change, so the set of concepts that we rely on, that we are happy to consider etc., will change.

Adaptivity seems to provide one such extension, and to point the way to which the general enactive framing will make possible specific kinds of scientific accounts within specific domains. The innovation of adaptivity is to regard one link in the chain of mediation as closed, by taking particular cybernetic norms as given, and in need of no further justification to a specific community of observers. In consideration of the metabolic sense-making activity of a lone cell, it is by no means difficult to assent to this, no more difficult, in fact, than to accept the role of the heart as a pump. But it is useful to recognize what we have done when we provide such assent. Joint recognition of the autopoietic nature of the cell was possible because we recognized it as a self-producing unity. Joint assent to the innovation of adaptivity is a further step, one that embeds all subsequent accounts that build on it within the frame of sense-making by the unity observed.

## 6. On Levels and Distinctions

Varela (1979) produced a formidable formalization of the enactive agenda in his Principles of Biological Autonomy. In that, he extended the foundational work of Spencer-Brown (1969) as laid out in his enigmatic and profound Laws of Form. Spencer-Brown tries to find a starting point for formal description that is prior to both mathematics and logic, and he does so by taking as given the idea of *distinction* and the idea of *indication*. A distinction, we might observe in the present context, is drawn by a community of observers when they pick out, by indication, something to characterize. The unity that is at the origin of the autopoietic account is distinguished by being so picked out. At the outset, Spencer-Brown says “There can be no distinction without motive, and there can be no motive unless the contents are seen to differ in value” (p. 1).

To embodied beings, the motive is clear. We recognize the cell as a minimal form of life, and we understand its sense-making as an activity that continuously produces the distinction between self and non-self. We value the living over the inert. This is the starting point for the enactive programme. It is not the starting point for all formal or informal discussion, but it is a principled starting point when we wish to progress to a scientific account that can be relevant to the lives of individual embodied beings.

Louis Kauffman describes the intertwining of the observer and the observed thus (Kauffman, 2017, p. 11):

An organism is seen, by an observer, to make a distinction. By starting with a distinction we understand how (for an observer) the organism exhibits structural stability and autonomy, and becomes an exemplar of the living. This notion of distinction is crucial to our understanding of the nature of an organism and the nature of life itself. The distinction is a joint creation of the organism in its environment and the observer. Together they give life to the organism. The distinction does not appear without the observer, and the distinction that is the organism does not appear without the actions of the organism, producing itself from itself through components taken from and given back to the environment.

The introduction of adaptivity in order to license the cybernetic machinery of the lonesome cell draws a further distinction. This too is motivated, but the motive is a step further than the indication of a distinguished dynamically persisting unity. One could draw further distinctions, for many reasons. It is a long way from the cell to any account of a multi-cellular body, and along the way, our accounts will involve the drawing of distinctions that might be contested. Distinctions drawn within one domain of discourse may be pursued and refined, while they may be never drawn in a separate discourse.

Within the calculus of indications developed by Spencer-Brown and used by Varela, we can draw further distinctions predicated upon any starting distinction, traveling down into a world of detail and structure. We can also undo distinctions, popping back up to higher levels, and allowing different subsequent courses of discrimination, with different commitments. This calculus formalizes the operations which are both kataphatic (drawing distinctions) and apophatic (undoing distinctions). If kataphasis produces positivist assertions, apophasis tentatively undoes prior distinctions, allowing us to regard the effect of such distinctions, and the landscape that opens up if we had chosen other distinctions and pursued other debates^5^. We can get a sense of how this applies to the discussion of the cell by contrasting the operational and observational levels of description.

At the operational level, the cell recursively produces itself. It does so with no reference to any environment. Environmental influences are seen as external perturbations, uninterpretable, though exerting influence on the processes of production. The components of the cell are taken as given and we pay attention to their mutual relations.

When we pull back and consider the cell in its environment, the cell, which was characterized as a suite of processes, is now cast in a different role, as a distinguished *thing* that exhibits a *behavior*, predicated upon inputs (glucose readings) and outputs (locomotory activity). Calling something a *behavior* is a cybernetic, purposive, characterization. It comes at the cost of simplifying the account of the cell, removing reference to recursive self production, and the important simplification that the outputs of the cell are assumed to simply land in the environment, without consequence for its characterization.

We can continue to pull back. To quote Varela once more:

The cell biologist emphasizes the cell's autonomy, and views the organism of which it is part as little more than a source of perturbations for which the cell compensates. But the physiologist views the cell as an element in a network of interdependences constituting the individual organism: This corresponds to a wider view of environment, namely the ecology in which the individual participates. A population biologist makes his distinctions at a still higher level, and largely ignores the cell. A similar hierarchy of levels can be found in the social sciences. It seems to be a general reflection of the richness of natural systems that indication can be iterated to produce a hierarchy of levels (Varela, 1979, p. 85/86).

Varela here might be misunderstood, and the misunderstanding can best be seen by returning to our opening theme of breaking the fourth wall, which can have two contrasting effects: widening the magic circle, or extinguishing the drama.

Widening the circle is analogous to the approach taken by Nagel to “objectivity”:

To acquire a more objective understanding of some aspect of life or the world, we step back from our initial view of it and form a new conception which has that view and its relation to the world as its object. In other words, we place ourselves in the world that is to be understood. The old view then comes to be regarded as an appearance, more subjective than the new view, and correctable or confirmable by reference to it. The process can be repeated, yielding a still more objective conception (Nagel, 1986, p. 4).

For Nagel, this process never stops. There is no view from nowhere that cannot be improved by pulling back, and forming a larger, more encompassing view that takes our position as observers into some account. The magic circle continues to expand, but the drama never stops either. It just gets bigger, as if all descriptive accounts could be accommodated within a single regime of truthful correspondence. The nature of the account at one level is also not fundamentally altered as we move up and down the levels. Introducing the hermeneutic reference to our own role as observers does not change the characterization of anything that went before. It may serve to relativize our previous account, but it does not fundamentally change it. To the extent that it admits of continued enlargement, this approach to scientific description is universalizing in its character, and in the asymptotic limit it would reach a picture of “nature” where all truths are written.

Latour, to whom this universalizing approach to nature is anathema, lampoons the idea of a universal regime of truth. In his 2013 Gifford Lectures on Natural Theology, he takes David Hume as his foil, and says:

For him, it seems, there is just one regime of truth that he may use exactly in the same fashion to ask his butler if he should carry an umbrella to visit his friend, Adam Smith, if his mistress loves him for good, if Cromwell was born on the 25th of April, 1599, or if God is a spider, an architect, or a giant vegetable (Latour, 2013).

Latour is, of course, more sensitive than most to the muddles that arise when we pretend that objective descriptive accounts can be cleanly separated from those that lean on function, purpose, or behavior. The sensitivity to the responsibility of the community who draw distinctions makes all such scientific work inherently political, and the reliance on indubitable commitments of a community of observers makes religious considerations a necessary part of ecological discussion too.

Varela is not relying on this kind of universalizing approach to “nature.” Here, we might return to the deflationary effect produced when breaking the fourth wall, as Brecht and Jodorowsky employ it. When we draw our attention to the role of the observers in drawing distinctions, we do not simply add the observer as another represented element within a somewhat enlarged discursive domain. Rather, it becomes clear that the observer community is always a participant in founding the epistemology. A participatory epistemology is revealed in which describer and described are interlocked. In Spencer-Brownian terms, we say that the distinction is reentered in the space of the observer, making those drawing the distinction responsible for the distinctions they draw. “Own your own distinctions!” is the injunction.

## 7. Ecological Psychology and Its Relation to Enaction

The field of ecological psychology seems to be poorly named. It is not concerned with most of the broad concerns that motivated the foundation of scientific psychology, often enumerated as experience and behavior. On the experience side of the account, it has nothing to say about phenomenology, experience, emotions, or feelings^6^. Two of the three central pillars that have enabled the construction of the psychological subject, perception, attention, and memory, are omitted altogether, while the third, perception, is redefined in relational terms, thereby radically transforming the notion of perception to something quite alien to all internalist theories. It typically addresses the control of behavior with minimal reliance on an executive controller. The term “ecological” is also unfortunate. It fails to take any engagement with ecology seriously as it inevitably assumes a physical environment as a given, and it ignores the interdependencies of multiple life forms. Perhaps a rebranding is in order?

Yet it has provided some of the most assured, insightful and powerful accounts within the domain of perceptually guided action. The old injunction to ask what the head is inside of, instead of what is inside the head, turns out to set an excellent working agenda, that can be tackled using clearly defined concepts, empirical variables that can be directly measured, and to produce insights about the fit between the capacities of an organism and its immediate physical surround. The technical innovation of the affordance allows such relations to be explored in functional terms, relative to the goal-directed behavior (Chemero, 2003).

To many researchers, most of the present contributors included, there seems to be something quite compatible between the subject-world relation explored reflexively within enaction and the organism-surround relations laid out and quantified within the Gibsonian frame. And yet nothing in the foregoing discussion would be at home in an ecological psychology account. Why?

Let us take, as an illustrative example, the famous account of diving gannets by Lee and Reddish (1981). The work is sufficiently well-known that we can skip almost all of the details, looking only at those aspects that speak to the ongoing discussion. In that work, the expanding pattern that must be present on the retina of a perpendicularly diving bird as it approaches the textured surface of the water was considered, and found to be highly informative. Specifically, the time to contact with the water was directly available in a variable τ, derived from the rate of expansion of inhomogeneous elements in the optical pattern on the retina. Recognizing that this valuable information is just there for the taking greatly transforms our understanding of what the bird is doing, and what part of that might be apportioned to internal mechanisms. Gannets must dive with outstretched wings, but must retract them before entering the water. The presence of information on the surface of the retina about time to contact allows us to vastly reduce the explanatory load on hypothetical internal mechanisms, requiring little more than a thresholding mechanism to trigger wing retraction. Without the insight provided by the relational analysis, one might be fooled into attributing all kinds of complex computations and discriminations to the bird's brain (Van Gelder, 1995). The analysis transforms our view of what is going on completely.

But note the frame that is assumed by this analysis. We have a bird and a physical environment, both of which are taken as given. We have a behavior that is also presumed, with no reference to the observer. Because all organisms must feed, the behavior seems uncontroversial, and in need of mechanical explanation. Indeed, mechanism seems to be the preferred type of explanation in much work within the field (Golonka and Wilson, 2019). To the extent that behavior and environment are characterized to the satisfaction of a community of observers, the Gibsonian analysis appears especially insightful. The relation often described as “perception-action” is given a quantifiable objective (in these particular parentheses) characterization.

But a bird is far more than a mere organism. The enactive use of the term organism is usually grounded in consideration of the minimal form of life, the cell. Calling something an organism leaves much unsaid, but the frame will license discussion of some specific kinds of behaviors common to all organisms: feeding, excreting, locomoting, and perhaps a few more. Where most scientific psychology is still lamentably prone to using normative and teleological constraints as if Vitruvian Man were the accepted norm for a human, we have no Vitruvian Organism we can rely on. Diving for fish, wing-spreading and retraction are not behaviors that are common to all organisms, but they make sense in the shared discursive frame of the diving gannet viewed by behavioral scientists. Other organisms might leave us less secure in our framing of behavior or environment. Microscopic marine monsters, eyelash mites, corals, these cannot be viewed in the same way as a diving bird or a playing human child.

When ecological psychology employs the term “organism,” I cannot escape the feeling that it is trying to get away from the commitments of most psychological frameworks, and to suggest allegiance to the world of broader biological accounts. This is, of course, entirely in the spirit of the enactive approach, but the pictures so drawn are specific kataphatic^7^ elaborations drawn as required by the consideration of specific behaviors recognized in advance. The ecological analysis starts by singling out a “behavior” to be characterized, by fixing the organism/animal/agent and the environment of relevance, and it builds its account from there. In so doing, it frequently has the result that much of the explanatory load normally consigned to hidden interiorities and brains is reduced, but not removed.

## 8. Attributing Minds, Souls, and More

The reflexive characteristic of a discourse within an enactive framework requires that we be explicit in our assumptions, in the distinctions we choose to draw, and their acceptance or contestation by those conducting the discussion. But no discourse can make all distinctions patent. No matter how fundamental the frame we carefully draw, it is always drawn within a larger, unwritten and unspoken frame. In Spencer-Brown's formalism this is referred to as the unwritten cross, assumed to accommodate those distinctions that do not need to be drawn, because they are uncontroversial (Spencer-Brown, 1969, p. 6).

The ecological psychologist typically leaves consideration of agency at the door. The task-centered nature of the description is assumed, as the structure of the task is probed. There is an unspoken presumption, therefore, that whatever else is necessary for more complete, or merely overlapping discourses, will be found elsewhere. In this way, a Gibsonian is not committed to any fixed view of mind, agency, or individual executive control. Different framings of different tasks will leave unaddressed commitments at the door.

In this respect, a Gibsonian account is once more entirely within the spirit of an enactive approach. When we move beyond the bare recognition of autopoietic self-production and self-distinction and consider the cell as a minimal sense-making form, we find we need further distinctions to conduct our account. This is where adaptivity is introduced, because it is needed. It is an add-on though, demanded of the specific example being discussed. This is how an enactive framing grows its distinctions within specific discourses, creating descriptive domains of increasing detail and complexity. Further attributions will be needed as more complex systems are considered. Each add-on might be contested by the observing collective, and the resulting detailed detailed descriptive characterization will depend on the consent of the collective to make sense. Objectivity in parentheses allows us to nest parentheses, but we need to clean up after ourselves, and not assume that distinctions drawn and accepted within one discourse can thereby be wielded without caution more generally.

The elephant in the room I am skirting around is the psychological subject, so central to most narrative accounts of the person. Centuries of debate, and 150 years of constructed methods and experimental vehicles have installed in most of our debates the idea that a person is a subject who perceives a world, allocating attentional resources, with access to a transcendental memory database, and ruled over by an executive controller. This is a being endowed with something called a “mind” and animated by a single spirit. Spelled out in this stark fashion, one might indeed begin to ask whether the distinctions drawn that are necessary to allow this descriptive figure to go unchallenged are either secure or might demand consent. If one addresses any of the central elements in this construction, they are easily seen to be subject to challenge, and they become entirely untrustworthy in any debate not framed by some kind of materialism. The Buddhist roots of much of the enactive approach are destabilizing precisely because they belong within a different metaphysical frame. Increasing awareness of the existence of Buddhist or Advaitic frameworks, in particular, might help to increase awareness that the dualities required to support the psychological subject are local in character, grew within a specific cultural and theological framework, and to encourage us to consider alternatives. There is not room here to pursue this in depth, but the foregoing discussion allows us to perhaps provide a weak pointer.

Enaction does not start with the posit of individual personal minds. This leaves us without many of the familiar constructs we lean on in our everyday discourse. The enactive framework is young, and developing. Cast as mind-in-life, it adopts a maximally consensual starting point, situating us, the discussants and observers, among the living. The leap of faith Varela calls us to is nothing more than the consensual adoption of this common ground. The elaboration of this basic discursive position allows us to construct and create many kinds of descriptive characterizations in a strictly scientific mode. Ecological psychology has provided us with one such example. There will be others, leaning on other frames of distinction. They will satisfy our need for explanation to the extent that the distinctions drawn are consensual, and can survive the objections.

For at the heart of the enactive move is not an act of description, but an act of recognition. In picking out the cell as a living agent, we recognize our embedding in a world from which we are not distinct. Even in the ecological characterization of the gannets, we find a familiar world of birds and surfaces. We are at home there, too, and we happily conduct our analysis with a background that attributes the required animating spirit to the bird, sufficient to take care of that which we did not get around to considering. The mind constructed by scientific psychology is an elaboration of the notion of the soul as a singular animating force (Reed, 1998), but we are multiply animated. The person is not a fixed entity, nor a mere organism, but a locus of mutual recognition and negotiation.

Zoom out camera!

## Author Contributions

The author confirms being the sole contributor of this work and has approved it for publication.

## Conflict of Interest

The author declares that the research was conducted in the absence of any commercial or financial relationships that could be construed as a potential conflict of interest.

## Footnotes

^1^The distinction between teleonomy and teleology is crucial here. Teleology is concerned with purposes that exist. Teleonomy is concerned with accounts that refer to purposes, in the understanding that such purposes do not really exist, but could be replaced, in principle if not in practice, by a more expansive account.

^2^More fulsome descriptions are available, e.g., in Di Paolo (2019, p. 5).

^3^The manner in which this distinction between the observational and operational domains is treated in the broad context of enaction is not always well-aligned with the similar distinctions drawn in the more specific context of autopoietic theory, and the reader is warned that my elaboration here straddles the two fields somewhat uneasily. Thank you to one of the reviewers for drawing my attention to this potential confusion.

^4^There are accounts of indexicality in which causality here is explicitly ruled out. I mean a one step overt chain of mediation, from pathology to spot, from face to cloth, as causal, mediated through direct contact or continuity, without any representational hiatus, and uncontested within the frame of a specific discourse.

^5^Iconoclasm seeks to disenchant by denying specific attributions; Apophasis is a rhetorical move, allowing consideration of alternative framings and starting points.

^6^My ecological colleagues will rightly disagree, as a great deal of recent work, e.g., by Rietfeld, Chemero, and others, significantly expands the scope of ecological accounts. Here, I draw this picture in broad and crude strokes, leaning on early canonical work that has come to partially define the field, and in that limited context, I believe, the field is built in terms foreign to most of the field of psychology.

^7^That is, built upon specific positive distinctions required to conduct the discourse in a manner intelligible to all discussants.
